# Development of mutation-selective LRRK2 kinase inhibitors as precision medicine for Parkinson's disease and other diseases for which carriers are at increased risk

**DOI:** 10.3389/fneur.2022.1016040

**Published:** 2022-10-26

**Authors:** Robert K. Lesniak, R. Jeremy Nichols, Thomas J. Montine

**Affiliations:** ^1^Medicinal Chemistry Knowledge Center, Sarafan Chemistry, Engineering and Medicine for Human Health, Stanford University, Stanford, CA, United States; ^2^Department of Pathology, Stanford University, Stanford, CA, United States

**Keywords:** LRRK2, kinase, selectivity, inhibitors, Parkinson's disease

## Introduction

Parkinson's disease (PD) was first recorded clinically in 1817, and today, we recognize both motor and non-motor symptoms as characteristic of PD. Unfortunately, to this day, treatments for PD have not generally progressed beyond the symptomatic, relieving symptoms but not targeting the underlying cause(s) of the disease. Furthermore, many currently administered PD therapies yield unwanted side effects. The global prevalence of PD (~10 million) is set to increase, and therefore so has the urgency to develop novel and effective treatments to slow or even reverse the progression of PD (1–3).

The classic neuropathologic features of PD are the degeneration of dopaminergic neurons in the substantia nigra pars compacta (SNpc) and the appearance of intraneuronal Lewy bodies, which are formed in part by aggregated pathologic forms of α-synuclein, a presynaptic protein. Although originally thought to have minimal genetic influence, research over the last two decades has established numerous genetic causes and risk loci for PD (4, 5). Of the genetic variants that have been associated with PD, the most common genetic cause of familial and sporadic PD is mutation in LRRK2 (PARK8) (6, 7). Clinically and pathologically, PD caused by LRRK2 mutations is largely indistinguishable from idiopathic PD, except for lower risk of cognitive impairment and greater variation in the type of intraneuronal inclusion.

**Figure 1:**
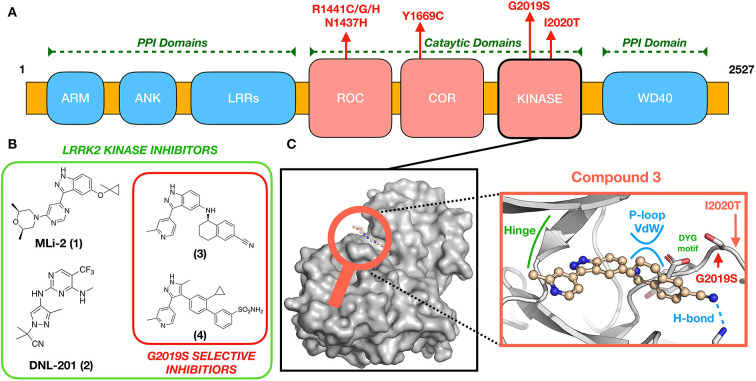
**(A)** LRRK2 domain organization and location of pathogenic mutations. **(B)** Chemical structures of some known brain-penetrant LRRK2 kinase inhibitors, including G2019S-selective compounds. **(C)** An expanded view (center) of (3) docked to a GS-LRRK2 kinase active site homology model (8), including locations of the known pathogenic mutations G2019S and I2020T. Compound 3 is proposed to interact uniquely with G2019S-LRRK2 through glycine-rich P-loop Van der Waals (vdw) and hydrogen bonding interactions.

Transcription and translation of LRRK2 yield a large (286 kDa) multi-domain protein, LRRK2, a member of the Roco superfamily of proteins. LRRK2 is composed of a tandem Ras complex (Roc) GTPase-domain linked to a kinase domain through a carboxy-terminal (COR) sequence (Figure 1A). Outside of the characteristic Roco family motifs, LRRK2 possesses four protein–protein interaction (PPI) domains: WD40, armadillo repeats (ARM), ankyrin repeats (ANK), and the namesake leucine-rich repeats (LRR). These domains likely participate in the regulation of LRRK2 localization (9–11) and LRRK2 kinase activity (WD40) (12) as well as mediate changes in structural conformation to active and inactive states (13–15). LRRK2 has been implicated in numerous cellular processes, including vesicle trafficking, cytoskeletal maintenance, and autophagy, reviewed in (16, 17). Although the precise physiological function(s) disrupted by LRRK2 missense mutations is yet to be identified, evidence to date suggests that altered activities of its enzymatic domains—kinase and ROC-GTPase—are key contributors (17, 18). These compelling genetic and biochemical data have led to the hypothesis that eliminating aberrant LRRK2 kinase or ROC-GTPase activity might be an effective therapeutic strategy for people with PD who carry LRRK2 missense mutations.

## Targeting the LRRK2 kinase domain

Several missense mutations of LRRK2 are dominantly inherited causes of PD, including G2019S, I2020T, R1441C/G/H, N1437H, and Y1699C, all of which occur in the catalytic domains of LRRK2 (Figure 1). The most prevalent, G2019S-LRRK2, accounts for 6% of familial and 2% of sporadic PD (19). By far, most people with PD from G2019S-LRRK2 have inherited one copy of the mutant allele, meaning that they also possess a wild-type (WT) LRRK2 allele. G2019S-LRRK2 also is reported to approximately double the risk of breast cancer in women (20, 21). Less common LRRK2 missense mutations also modulate kinase activity of LRRK2 by varying amounts (22–24); these represent but a few of the almost 100 known LRRK2 mutations, some of which also modulate risk of a form of inflammatory bowel disease and a form of leprosy (25).

G2019S-LRRK2 appears in the kinase domain (Figure 1A), specifically the DYG regulatory motif of the activation loop. As a result, G2019S-LRRK2 possesses hyperactive kinase activity defined by increased K_cat_ (but not V_max_) when compared to wild-type (WT) LRRK2 (22). Altered LRRK2 kinase activity is reliably observed in cells vs. *in vitro* (24, 26). The most validated substrates of LRRK2 include several Rab GTPase family members (24). Rab GTPases regulate the flow of vesicular traffic within the cell, and their phosphoregulation by LRRK2 modifies their membrane association and function (27). Increased kinase activity by mutant LRRK2 results in hyperphosphorylation and dysregulation of Rab substrates, which can be resolved by inhibition of LRRK2 kinase activity (24, 26). Lysosomal disruption and altered protein homeostasis, as a result, is a major proposed mechanism of Parkinson's disease pathogenesis (28–31). The effect of LRRK2 activity in idiopathic Parkinson's disease is only now becoming understood by examining post-mortem tissue as well as patient blood, urine, and CSF samples (32, 33). Release of the lipid BMP (22:6/22:6), an indicator of lysosome dysfunction, is increased in rodents and patients harboring the LRRK2-GS mutation and is reduced in LRRK2 knockout rodents, LRRK2 inhibitor treated non-human primates, and patients treated with DNLI-151/BIIB122 (8, 30, 34). These data suggest a connection between lysosome function and LRRK2 kinase activity. BMP levels and phosphoRab levels appear to be related and reliable markers of LRRK2 inhibition for clinical trials. These, along with the robust LRRK2 pSer935 upstream kinase phosphorylation site (35), make pharmacodynamic detection of target engagement in animals and humans possible (36).

Substantial PD drug discovery efforts over the last two decades have focused on the development of brain-penetrant LRRK2 kinase inhibitors, yielding multiple small molecules fit for this purpose. Notable and efficacious examples of LRRK2 inhibitors widely deployed in the field include MLi-2 (1), DNL-201 (previously known as GNE-0877) (2), and PF-360 (not shown) developed by Merck (37), Genentech/Denali (38), and Pfizer (39), respectively (Figure 1B). Despite these successes in medicinal chemistry, few lead compounds have reached clinical trials, often because of initial safety concerns (8).

Typically, kinase inhibitor programs geared toward cancer therapeutics involve short-term doses until remission of tumors. The envisioned medical management of LRRK2-GS carriers would be different. In LRRK2-GS Parkinson's disease, there is a long preclinical stage where a carrier could be identified by genetic testing decades before he or she was symptomatic. Transition to a prodromal stage might be estimated by family history of age of onset or assessed by changes in biomarker or neuroimaging screening that signify brain injury and herald the pending onset of the clinical stage of Parkinson's disease. Ideally, treatment with kinase inhibitors would be considered at the prodromal stage or perhaps earlier, balancing the benefits of starting treatment before onset of symptoms against the risk of likely treatment for the remainder of one's life. Indeed, in this scenario, exposure to drug may include decades of an older individual life, highlighting the importance of safety and tolerability (40).

Phase 1b clinical trials of two candidates, DNL-201 (**2**) and DNL-151/BIIB122 (a likely structurally related type I, ATP-competitive kinase inhibitor), reported that short-term administration did not cause adverse events (30, 41). Of the two, DNL-151/BIIB122 was selected to progress into further clinical trials due to its preferred pharmacokinetic properties (30, 42). The clinical trial NCT05348785 sponsored by Biogen with their collaborator Denali will test BIIB122 in a phase 2b multicenter, randomized, double-blind, placebo-controlled study to determine the efficacy and safety in participants with early clinical stage PD (30–80 years old). BIIB122 will also be assessed in a Phase 3 clinical trial NCT05418673, a multicenter, randomized, double-blind, placebo-controlled study of symptomatic Parkinson's disease patients carrying the LRRK2-GS mutation.

It is important to note that the compounds being tested in the above clinical trials are non-selective LRRK2 kinase inhibitors, equivalently inhibiting both mutant and WT kinase and potentially causing untoward on-target effects from suppression of WT kinase activity. Most preclinical data indicate that pharmacologic inhibition of LRRK2 kinase results in pathologic changes to the lung in rodents and non-human primates; however, no associated changes in respiratory function were observed. Furthermore, the major structural change in the lung, hypertrophy of type II pneumocytes, washed out after drug withdrawal. Clinical trials of DNLI-201 NCT03710707 and DNL-151/BIIB122 NCT04056689 included pulmonary monitoring and observed no adverse events in the time frame of the trial (30). It remains to be determined in animal models and humans if all pathologic changes in the lung revert upon drug withdrawal, especially after prolonged exposure.

In an effort to avoid these potential on-target side effects, we and others recently have undertaken the development of brain-penetrant, highly selective G2019S-LRRK2 kinase inhibitors to test the hypothesis that such precision therapeutics might bring similar benefits with less side effects—especially when administered over years to decades—to people with PD driven by G2019S-LRRK2 (43–46). Moreover, related non-brain-penetrant inhibitors might find application in G2019S-LRRK2 mutation carriers at increased risk for breast cancer as a potential anti-cancer agent acting solely in the periphery.

## Precision medicine for PD—Targeting the G2019S-LRRK2 kinase domain

From a medicinal chemistry perspective, design of a G2019S-selective kinase inhibitor would seem exceedingly challenging as a single amino acid differentiates WT from G2019S-LRRK2. This would suggest that the kinase domain of the two variants would be near identical. Indeed, recent Cryo-EM data support this assumption, with the authors suggesting that hyperactive G2019S-LRRK2 kinase activity may be kinetic in nature rather than structural (47). In contrast to these findings, kinetic and computational studies involving type II kinase inhibitors (preferentially binding the DYG-out, “inactive” kinase conformation) show LRRK2 activation loop variants, that is, G2019S and I2020T, more readily stabilize the “active,” DYG-in kinase conformation in solution when compared to WT-LRRK2 and thus do not bind type II kinase inhibitors effectively. This observation was rationalized with computational modeling, revealing Ser2019 of G2019S-LRRK2 directly forms an H-bonding network in the kinase active site involving key catalytic residues (48, 49). If indeed G2019S-LRRK2 exists as DYG-in stabilized in solution, then the kinase domain architecture may be distinctly different to that of WT-LRRK2, which can readily access both DYG-in and DYG-out conformations. Interestingly, crystallographic surrogates (CHK1 mutants) of the G2019S-LRRK2 kinase active site revealed a DYG-in conformation, at least in the presence of type I kinase inhibitors. Such CHK1-LRRK2 surrogates have been used effectively in the pursuit of novel LRRK2 kinase inhibitors, although these were not disclosed as G2019S-selective (50–52). In the design of G2019S-selective compounds, the situation is less clear.

Although molecular modeling efforts based on such G2019S-LRRK2 surrogates have resulted in extremely selective compounds, such as (3) (44) and (4) (45), selectivity toward G2019S appears to be amplified in the whole organism (43–45); thus, specific assay techniques may be required to ascertain true compound selectivity. The combined forces of molecular modeling, screening, and iterative compound synthesis have allowed for the successful design of selective G2019S-LRRK2 kinase inhibitor(44, 45). Thus, far, the structure of published selective G2019S-LRRK2 compounds supports the aforementioned hypothesis established using type II compounds (48, 49) that G2019S kinase provides a unique binding site, not accessible in WT-LRRK2, where Van der Waals interactions are possible between appropriately designed small molecules and the G2019S kinase glycine-rich loop (Figure 1C) (44, 45). Beyond the aforementioned challenges in designing G2019S-LRRK2 selective kinase inhibitors, it is an essential requirement for a successful PD therapeutic to be brain-penetrant; few reported examples of these exist, for example (3) (44). However, highly selective G2019S-LRRK2 kinase inhibitors that do not efficiently access the brain may find applications in other diseases to which mutation carriers have increased risk, such as breast cancer.

## Conclusion

The preponderance of evidence has identified the G2019S-LRRK2 hyperactive kinase function as pathogenic in the context of PD. Searching for inhibitors of this enzymatic function should therefore be considered a worthwhile target that may bring benefit to G2019S-LRRK2 carriers in the first instance, with the potential for downstream therapeutic benefits as well. At the very least, a clinically useful G2019S-selective, brain-penetrant inhibitor could be used to elucidate the complex molecular biology of mutant LRRK2 and thus be enormously beneficial to PD precision medicine development. How this mutation affects LRRK2 the cellular functions remains unclear. To date, we have observed that this single amino acid mutation creates a structural effect impacting kinase kinetics, conformation, and substrate preference. The challenges involved in developing a selective G2019S-LRRK2 kinase inhibitor perhaps have led to more emphasis on non-selective inhibitor development, even while questions still remain regarding the safety and efficacy of non-specific LRRK2 inhibitors. As described herein, data-driven approaches utilizing both computational modeling and medicinal chemistry have overcome some of these challenges to identify potent, selective, and brain-penetrant G2019S-selective kinase inhibitors. Ultimately, G2019S-LRRK2 has been shown to be a valid therapeutic target, with established links to PD pathogenesis and extensive evidence suggesting inhibitors of the mutant LRRK2 kinase domain may yield powerful, precision therapeutics. As the first LRRK2 kinase inhibitors (non-selective for genetic variants) are progressing through clinical trials, time will tell whether such compounds are able to fulfill the unmet medical need for safe and effective PD treatments. Including more precise, mutant-selective LRRK2 inhibitors to our arsenal will potentially provide safer medications for mutation carriers who face an increased risk of PD and beyond.

## Author contributions

RKL, RJN, and TJM: figure design, interpretation of data, drafting the submitted material, and critical review. All authors contributed to the article and approved the submitted version.

## Funding

This study was funded by the Alexander & Eva Nemeth Foundation, the Sergey Brin Family Foundation, and the Farmer Family Foundation Parkinson's Research Initiative.

## Conflict of interest

The authors declare that the research was conducted in the absence of any commercial or financial relationships that could be construed as a potential conflict of interest.

## Publisher's note

All claims expressed in this article are solely those of the authors and do not necessarily represent those of their affiliated organizations, or those of the publisher, the editors and the reviewers. Any product that may be evaluated in this article, or claim that may be made by its manufacturer, is not guaranteed or endorsed by the publisher.

## References

[1] Marras C Beck JC Bower JH Roberts E Ritz B Ross GW. Prevalence of Parkinson's disease across North America. NPJ Parkinsons Dis. (2018) 4:21. 10.1038/s41531-018-0058-0 30003140 PMC6039505

[2] Pringsheim T Jette N Frolkis A Steeves TD. The prevalence of Parkinson's disease: a systematic review and meta-analysis. Mov Disord. (2014) 29:1583–90. 10.1002/mds.25945 24976103

[3] Dorsey ER Constantinescu R Thompson JP Biglan KM Holloway RG Kieburtz K. Projected number of people with Parkinson disease in the most populous nations, 2005 through 2030. Neurology. (2007) 68:384–6. 10.1212/01.wnl.0000247740.47667.03 17082464

[4] Nalls MA Plagnol V Hernandez DG Sharma M Sheerin UM Saad M. Imputation of sequence variants for identification of genetic risks for Parkinson's disease: a meta-analysis of genome-wide association studies. Lancet. (2011) 377:641–9. 10.1016/S0140-6736(10)62345-8 21292315 PMC3696507

[5] Nalls MA Blauwendraat C Vallerga CL Heilbron K Bandres-Ciga S Chang D. Identification of novel risk loci, causal insights, and heritable risk for Parkinson's disease: a meta-analysis of genome-wide association studies. Lancet Neurol. (2019) 18:1091–102. 10.1016/S1474-4422(19)30320-5 31701892 PMC8422160

[6] Zimprich A Biskup S Leitner P Lichtner P Farrer M Lincoln S. Mutations in LRRK2 cause autosomal-dominant parkinsonism with pleomorphic pathology. Neuron. (2004) 44:601–7. 10.1016/j.neuron.2004.11.005 15541309

[7] Paisán-Ruíz A Jain S Evans EW Gilks WP Simón J van der Brug M. Cloning of the gene containing mutations that cause PARK8-linked Parkinson's disease. Neuron. (2004) 44:595–600. 10.1016/j.neuron.2004.10.023 15541308

[8] Fuji RN Flagella M Baca M Brodbeck J Chan BK Fiske BK. Effect of selective LRRK2 kinase inhibition on nonhuman primate lung. Sci Transl Med. (2015) 7:273ra15. 10.1126/scitranslmed.aaa3634 25653221

[9] Purlyte E Dhekne HS Sarhan AR Gomez R Lis P Wightman M. Rab29 activation of the Parkinson's disease-associated LRRK2 kinase. EMBO J. (2018) 37:1–18. 10.15252/embj.201798099 29212815 PMC5753036

[10] Vides EG Adhikari A Lis P Purlyte E Shumate J Lasso ES. A feed-forward pathway drives LRRK2 kinase membrane recruitment and apparent activation. BioRxiv. (2022) 2022:489459. 10.1101/2022.04.25.489459 PMC9576273 36149401

[11] Zhu H Tonelli F Alessi DR Sun J. Structural basis of human LRRK2 membrane recruitment and activation. BioRxiv. (2022) 2022:489605. 10.1101/2022.04.26.489605

[12] Zhang P Fan Y Ru H Wang L Magupalli VG Taylor SS. Crystal structure of the WD40 domain dimer of LRRK2. Proc Natl Acad Sci USA. (2019) 116:1579–84. 10.1073/pnas.1817889116 30635421 PMC6358694

[13] Deniston CK Salogiannis J Mathea S Snead DM Lahiri I Matyszewski M. Structure of LRRK2 in Parkinson's disease and model for microtubule interaction. Nature. (2020) 588:344–9. 10.1038/s41586-020-2673-2 32814344 PMC7726071

[14] Watanabe R Buschauer R Bohning J Audagnotto M Lasker K Lu TW. The *in situ* structure of Parkinson's disease-linked LRRK2. Cell. (2020) 182:1508–18 e16. 10.1016/j.cell.2020.08.004 32783917 PMC7869717

[15] Guaitoli G Raimondi F Gilsbach BK Gomez-Llorente Y Deyaert E Renzi F. Structural model of the dimeric Parkinson's protein LRRK2 reveals a compact architecture involving distant interdomain contacts. Proc Natl Acad Sci USA. (2016) 113:E4357–66. 10.1073/pnas.1523708113 27357661 PMC4968714

[16] Bonet-Ponce L Cookson MR. LRRK2 recruitment, activity, and function in organelles. FEBS J. (2021) 2021:16099. 10.1111/febs.16099 34196120 PMC8744135

[17] Alessi DR Sammler E. LRRK2 kinase in Parkinson's disease. Science. (2018) 360:36–7. 10.1126/science.aar5683 29622645

[18] Park Y Liao J Hoang Q Roc Q. The G-domain of the Parkinson's disease-associated protein LRRK2. Trends Biochem Sci. (2022) 6:9. 10.1016/j.tibs.2022.06.009 35840518 PMC9669111

[19] Kay DM Zabetian CP Factor SA Nutt JG Samii A Griffith A. Parkinson's disease and LRRK2: frequency of a common mutation in US movement disorder clinics. Mov Disord. (2006) 21:519–23. 10.1002/mds.20751 16250030

[20] Agalliu I San Luciano M Mirelman A Giladi N Waro B Aasly J. Higher frequency of certain cancers in LRRK2 G2019S mutation carriers with Parkinson disease: a pooled analysis. J Am Med Assoc Neurol. (2015) 72:58–65. 10.1001/jamaneurol.2014.1973 25401981 PMC4366130

[21] Parrilla Castellar ER Plichta JK Davis R Gonzalez-Hunt C Sanders LH. Somatic mutations in LRRK2 identify a subset of invasive mammary carcinomas associated with high mutation burden. Am J Pathol. (2020) 190:2478–82. 10.1016/j.ajpath.2020.08.010 32931768

[22] Jaleel M Nichols RJ Deak M Campbell DG Gillardon F Knebel A. LRRK2 phosphorylates moesin at threonine-558: characterization of how Parkinson's disease mutants affect kinase activity. Biochem J. (2007) 405:307–17. 10.1042/BJ20070209 17447891 PMC1904520

[23] West AB Moore DJ Choi C Andrabi SA Li X Dikeman D Biskup S. Parkinson's disease-associated mutations in LRRK2 link enhanced GTP-binding and kinase activities to neuronal toxicity. Hum Mol Genet. (2007) 16:223–32. 10.1093/hmg/ddl471 17200152

[24] Steger M Tonelli F Ito G Davies P Trost M Vetter M. Phosphoproteomics reveals that Parkinson's disease kinase LRRK2 regulates a subset of Rab GTPases. Elife. (2016) 5:12813. 10.7554/eLife.12813 26824392 PMC4769169

[25] Blauwendraat C Nalls MA Singleton AB. The genetic architecture of Parkinson's disease. Lancet Neurol. (2020) 19:170–8. 10.1016/S1474-4422(19)30287-X 31521533 PMC8972299

[26] Kalogeropulou AF Purlyte E Tonelli F Lange SM Wightman M Prescott AR. Impact of 100 LRRK2 variants linked to Parkinson's disease on kinase activity and microtubule binding. Biochem J. (2022) 479:1759–83. 10.1042/BCJ20220161 35950872 PMC9472821

[27] Pfeffer SR. LRRK2 phosphorylation of Rab GTPases in Parkinson's disease. FEBS Lett. (2022) 1–8. 10.1002/1873-3468.14492. [Epub ahead of print]. 36114007

[28] Root J Merino P Nuckols A Johnson M Kukar T. Lysosome dysfunction as a cause of neurodegenerative diseases: lessons from frontotemporal dementia and amyotrophic lateral sclerosis. Neurobiol Dis. (2021) 154:105360. 10.1016/j.nbd.2021.105360 33812000 PMC8113138

[29] Erb ML Moore DJ. LRRK2 and the endolysosomal system in Parkinson's disease. J Parkinson's Dis. (2020) 10:1271–91. 10.3233/JPD-202138 33044192 PMC7677880

[30] Jennings D Huntwork-Rodriguez S Henry AG Sasaki JC Meisner R Diaz D. Preclinical and clinical evaluation of the LRRK2 inhibitor DNL201 for Parkinson's disease. Sci Transl Med. (2022) 14:eabj2658. 10.1126/scitranslmed.abj2658 35675433

[31] Udayar V Chen Y Sidransky E Jagasia R. Lysosomal dysfunction in neurodegeneration: emerging concepts and methods. Trends Neurosci. (2022) 45:184–99. 10.1016/j.tins.2021.12.004 35034773 PMC8854344

[32] Loeffler DA Aasly JO LeWitt PA Coffey MP. What have we learned from cerebrospinal fluid studies about biomarkers for detecting LRRK2 Parkinson's disease patients and healthy subjects with Parkinson's-associated LRRK2 mutations? J Parkinson's Dis. (2019) 9:467–88. 10.3233/JPD-191630 31322581 PMC6700639

[33] Rideout HJ Chartier-Harlin MC Fell MJ Hirst WD Huntwork-Rodriguez S Leyns EGC. The current state-of-the art of LRRK2-based biomarker assay development in Parkinson's disease. Front Neurosci. (2020) 14:865. 10.3389/fnins.2020.00865 33013290 PMC7461933

[34] Alcalay RN Hsieh F Tengstrand E Padmanabhan S Baptista M Kehoe C. Higher urine bis(monoacylglycerol)phosphate levels in LRRK2 G2019S mutation carriers: implications for therapeutic development. Mov Disord. (2020) 35:134–41. 10.1002/mds.27818 31505072 PMC6981003

[35] Dzamko N Deak M Hentati F Reith AD Prescott AR Alessi DR. Inhibition of LRRK2 kinase activity leads to dephosphorylation of Ser(910)/Ser(935), disruption of 14-3-3 binding and altered cytoplasmic localization. Biochem J. (2010) 430:405–13. 10.1042/BJ20100784 20659021 PMC3631100

[36] Kelly K West AB. Pharmacodynamic biomarkers for emerging LRRK2 therapeutics. Front Neurosci. (2020) 14:807. 10.3389/fnins.2020.00807 32903744 PMC7438883

[37] Scott JD DeMong DE Greshock TJ Basu K Dai X Harris J. Discovery of a 3-(4-Pyrimidinyl) Indazole (MLi-2), an orally available and selective leucine-rich repeat kinase 2 (LRRK2) inhibitor that reduces brain kinase activity. J Med Chem. (2017) 60:2983–92. 10.1021/acs.jmedchem.7b00045 28245354

[38] Estrada AA Liu X Baker-Glenn C Beresford A Burdick DJ Chambers M. Discovery of highly potent, selective, and brain-penetrable leucine-rich repeat kinase 2 (LRRK2) small molecule inhibitors. J Med Chem. (2012) 55:9416–33. 10.1021/jm301020q 22985112

[39] Andersen MA Christensen KV Badolo L Smith GP Jeggo R Jensen PH. Parkinson's disease-like burst firing activity in subthalamic nucleus induced by AAV-α-synuclein is normalized by LRRK2 modulation. Neurobiol Dis. (2018) 116:13–27. 10.1016/j.nbd.2018.04.011 29680709

[40] von Linstow CU Gan-Or Z Brundin P. Precision medicine in Parkinson's disease patients with LRRK2 and GBA risk variants - let's get even more personal. Transl Neurodegener. (2020) 9:39. 10.1186/s40035-020-00218-x 33066808 PMC7565766

[41] ClinicalTrials. Study to Evaluate DNL201 in Subjects With Parkinson's Disease. ClinicalTrials.gov.

[42] Woscholski R Parker PJ. Inositol lipid 5-phosphatases–traffic signals and signal traffic. Trends Biochem Sci. (1997) 22:427–31. 10.1016/S0968-0004(97)01120-1 9397684

[43] Garofalo AW Bright J De Lombaert S Toda AMA Zobel K Andreotti D. Selective inhibitors of G2019S-LRRK2 kinase activity. J Med Chem. (2020) 63:14821–39. 10.1021/acs.jmedchem.0c01243 33197196

[44] Leśniak RK Nichols RJ Schonemann M Zhao J Gajera CR Fitch WL. Discovery of G2019S-selective leucine rich repeat protein kinase 2 inhibitors with *in vivo* efficacy. Eur J Med Chem. (2022) 229:114080. 10.1016/j.ejmech.2021.114080 34992038

[45] Lesniak RK Nichols RJ Schonemann M Zhao J Gajera CR Lam G. Discovery of 1H-pyrazole biaryl sulfonamides as novel G2019S-LRRK2 kinase inhibitors. ACS Med Chem Lett. (2022) 13:981–8. 10.1021/acsmedchemlett.2c00116 35707141 PMC9190033

[46] Lesniak RK Nichols RJ Smith M Montine TJ. Targeting LRRK2 mutations in Parkinson's disease. Future Med Chem. (2022) 2022:102. 10.4155/fmc-2022-0102 35730403

[47] Myasnikov A Zhu H Hixson P Xie B Yu K Pitre A. Structural analysis of the full-length human LRRK2. Cell. (2021) 184:3519–27 e10. 10.1016/j.cell.2021.05.004 34107286 PMC8887629

[48] Liu M Bender SA Cuny GD Sherman W Glicksman M Ray SS. Type II kinase inhibitors show an unexpected inhibition mode against Parkinson's disease-linked LRRK2 mutant G2019S. Biochemistry. (2013) 52:1725–36. 10.1021/bi3012077 23379419 PMC3966205

[49] Ray S Bender S Kang S Lin R Glicksman MA Liu M. The Parkinson disease-linked LRRK2 protein mutation I2020T stabilizes an active state conformation leading to increased kinase activity. J Biol Chem. (2014) 289:13042–53. 10.1074/jbc.M113.537811 24695735 PMC4036318

[50] Williamson DS Smith GP Acheson-Dossang P Bedford ST Chell V Chen IJ. Design of leucine-rich repeat kinase 2 (LRRK2) inhibitors using a crystallographic surrogate derived from checkpoint kinase 1 (CHK1). J Med Chem. (2017) 60:8945–62. 10.1021/acs.jmedchem.7b01186 29023112

[51] Williamson DS Smith GP Mikkelsen GK Jensen T Acheson-Dossang P Badolo L. Design and synthesis of Pyrrolo[2,3-d]pyrimidine-derived leucine-rich repeat kinase 2 (LRRK2) inhibitors using a checkpoint kinase 1 (CHK1)-derived crystallographic surrogate. J Med Chem. (2021) 64:10312–32. 10.1021/acs.jmedchem.1c00720 34184879

[52] Keylor MH Gulati A Kattar SD Johnson RE Chau RW Margrey KA. Structure-guided discovery of aminoquinazolines as brain-penetrant and selective LRRK2 inhibitors. J Med Chem. (2022) 65:838–56. 10.1021/acs.jmedchem.1c01968 34967623

